# Perinatal Environmental Tobacco Smoke Exposure in Rhesus Monkeys: Critical Periods and Regional Selectivity for Effects on Brain Cell Development and Lipid Peroxidation

**DOI:** 10.1289/ehp.8286

**Published:** 2005-09-07

**Authors:** Theodore A. Slotkin, Kent E. Pinkerton, Frederic J. Seidler

**Affiliations:** 1 Department of Pharmacology and Cancer Biology, Duke University Medical Center, Durham, North Carolina, USA; 2 Center for Health and the Environment, and California National Primate Research Center, University of California, Davis, California, USA

**Keywords:** β-adrenergic receptor, brain development, environmental tobacco smoke, heart development, lipid peroxidation, muscarinic acetylcholine receptor, nicotine

## Abstract

Perinatal environmental tobacco smoke (ETS) exposure in humans elicits neurobehavioral deficits. We exposed rhesus monkeys to ETS during gestation and through 13 months postnatally, or postnatally only (6–13 months). At the conclusion of exposure, we examined cerebrocortical regions and the midbrain for cell damage markers and lipid peroxidation. For perinatal ETS, two archetypal patterns were seen in the various regions, one characterized by cell loss (reduced DNA concentration) and corresponding increases in cell size (increased protein/DNA ratio), and a second pattern suggesting replacement of larger neuronal cells with smaller and more numerous glia (increased DNA concentration, decreased protein/DNA ratio). The membrane/total protein ratio, a biomarker of neurite formation, also indicated potential damage to neuronal projections, accompanied by reactive sprouting. When ETS exposure was restricted to the postnatal period, the effects were similar in regional selectivity, direction, and magnitude. These patterns resemble the effects of prenatal nicotine exposure in rodent and primate models. Surprisingly, perinatal ETS exposure reduced the level of lipid peroxidation as assessed by the concentration of thiobarbituric acid reactive species, whereas postnatal ETS did not. The heart, a tissue that, like the brain, has high oxygen demand, displayed a similar but earlier decrease (2–3 months) in lipid peroxidation in the perinatal exposure model, whereas values were reduced at 13 months with the postnatal exposure paradigm. Our results provide a mechanistic connection between perinatal ETS exposure and neurobehavioral anomalies, reinforce the role of nicotine in these effects, and buttress the importance of restricting or eliminating ETS exposure in young children.

Environmental tobacco smoke (ETS) exposure is now recognized as a health risk for pregnant women and children (Dunn and Zeise 1997; Witschi et al. 1997), and it is increasingly evident that ETS affects the developing brain and cardiovascular system (Eskenazi and Trupin 1995; Hutchison et al. 1998; Makin et al. 1991). The consequences of fetal or early neonatal ETS exposure mimic those of active maternal smoking, albeit with a lesser magnitude (Makin et al. 1991), and for both active smoking and ETS, the dose–effect relationships correlate well with the levels of nicotine and its metabolites (Fried et al. 1995). ETS generally achieves fetal nicotine metabolite concentrations similar to those seen with light, active maternal smoking (Eliopoulos et al. 1996; Jauniaux et al. 1999; Ostrea et al. 1994), and young children exposed to ETS typically display levels exceeding those seen in older children (Fried et al. 1995; Kohler et al. 1999).

Animal studies demonstrate conclusively that nicotine damages the developing brain by altering the formation, survival, and differentiation of brain cells, eliciting deficits in structure, synaptic function, and behavioral performance (Levin and Slotkin 1998; Slotkin 1998, 2004; Walker et al. 1999). This provides a mechanistic link between maternal smoking during pregnancy and adverse neurobehavioral consequences in the offspring (Fried et al. 1992, 1998, 2003; Wakschlag et al. 2002; Weitzman et al. 2002). However, much less is known about the mechanisms underlying comparable effects of ETS. In a recent pair of studies, we found that perinatal ETS exposure in rhesus monkeys elicits alterations in cell signaling in the developing brain akin to those identified for nicotine administration in rodents, including the up-regulation of nicotinic cholinergic receptors, a characteristic of chronic nicotine-induced neuronal stimulation (Slotkin et al. 2000, 2002). These findings were important for two reasons: first, they provided the first evidence that ETS supplies sufficient nicotine to the developing brain to evoke inappropriate activation of the pathways that lead to altered cell development, and second, they demonstrated these effects in primates. The latter point is particularly important: the rat and mouse are altricial species, so brain development at birth corresponds to fetal stages of human development (Rodier 1988), and thus the concentrations or temporal factors for nicotine or ETS may not reflect those experienced in typical human exposure scenarios.

The present study, again using rhesus monkeys, was undertaken for four distinct purposes. First, we examined the relative importance of continuous perinatal ETS exposure compared with later exposure, determinations that are essential to identify the critical periods in which the developing brain is vulnerable to adverse effects of ETS. Our earlier work in rats indicated an extended period of vulnerability, lasting well into the postnatal period, and therefore in the present study we examined perinatal exposure up to 13 months of age, compared with ETS administered only during later postnatal stages, from 6 through 13 months. Second, we examined a variety of cortical brain regions and the midbrain, areas that, based on the known effects of nicotine, are likely to be compromised by developmental ETS exposure (Levin and Slotkin 1998; Slotkin 1998, 2004). Third, within each region, we characterized the neural cell damage caused by the different ETS regimens, using strategies adapted from prior rodent studies of nicotine or ETS (Gospe et al. 1996; Levin and Slotkin 1998; Slotkin 1998, 2004). Each neural cell contains only a single nucleus (Winick and Noble 1965), so the DNA concentration (DNA per unit tissue weight) reflects the cell packing density (Bell et al. 1987; Slotkin et al. 1984; Winick and Noble 1965). We also characterized the complement of cell proteins that reflect indices of cell type and size. The brain contains numerous glia, which are considerably smaller than neurons and thus possess less total protein per cell and a higher surface-to-volume ratio, which can be assessed by the proportions of total protein/DNA and membrane/total protein. At the same time, as neurons specialize, they enlarge and develop axonal and neuritic projections, which increases both the total protein/DNA ratio and membrane/total protein ratio in parallel (Qiao et al. 2003, 2004; Slotkin et al. 2005). These indices thus provide insight into the architectural alterations underlying the neurochemical effects of ETS. For example, the typical response to neuronal injury, neuronal replacement by smaller and more numerous glia (O’Callaghan 1988, 1993), produces an increase in cell packing density, a decrease in total protein/DNA, and an increase in membrane/total protein. In contrast, neuronal loss accompanied by perikaryal swelling, another archetypical injury response (Roy et al. 2005), elicits a decrease in cell packing density, an increase in total protein/DNA, and a decrease in the membrane/total protein ratio. A third pattern, damage to neuritic projections, produces a decrement in the membrane/total protein ratio in the nerve terminal region but an increase in areas where reactive sprouting takes place (Kostrzewa and Jacobowitz 1974; Navarro et al. 1988).

As a fourth objective, we made determinations of lipid peroxidation. Nicotine induces free radical generation and contributes a major proportion of the net oxidative stress imposed by tobacco use (Bhagwat et al. 1998; Newman et al. 2002; Qiao et al. 2005; Yildiz et al. 1999). At the same time, many other products in tobacco smoke similarly have the potential to produce oxidative damage (Huang et al. 2005), and oxidative stress contributes to the effects of many neurotoxicants (Gitto et al. 2002; Gupta 2004; Ohtsuka and Suzuki 2000; Olanow and Arendash 1994). To evaluate the role of oxidative damage in the effects of ETS in our primate model of brain development, we assessed the concentration of thiobarbituric acid–reactive species (TBARS) (Guan et al. 2003), contrasting the effects in brain regions with those in the heart. Both the brain and heart are highly vulnerable because of their high oxygen consumption, but the brain is especially sensitive for two reasons: first, neural cell membrane lipids are high in oxidizable polyunsaturated fatty acids (Gupta 2004); second, the developing brain has an increased metabolic demand associated with its perinatal growth spurt, during which it has lower reserves of protective enzymes and antioxidants (James et al. 2005) and is deficient in glia, which ordinarily protect neurons from oxidative molecules (Tanaka et al. 1999). In addition, we conducted studies to characterize the temporal appearance of alterations in cardiac TBARS as well as the potential neurotransmitter-receptor–driven mechanisms that underlie developmental vulnerability or protection from oxidative stress.

## Materials and Methods

### Materials.

We purchased standardized 1R4F research cigarettes from the University of Kentucky (Louisville, KY). [^3^H]AFDX384 (specific activity, 133 Ci/mmol) and [^125^I]iodo-pindolol (specific activity, 2,200 Ci/mmol) were obtained from PerkinElmer Life Sciences (Boston, MA). All other chemicals were purchased from Sigma Chemical Co. (St. Louis, MO).

### Animal treatments.

All studies were carried out in accordance with the declaration of Helsinki and with the *Guide for the Care and Use of Laboratory Animals* as adopted and promulgated by the National Institutes of Health (National Research Council 1996). We obtained 15 pregnant rhesus macaque monkeys from the California National Primate Research Center breeding colony and assigned them to three different treatment groups: animals to be exposed to filtered air, those to receive both prenatal and postnatal ETS exposure, and those to receive postnatal exposure only. The estimated gestational age for each dam was established by sonography performed before gestation day (GD) 40. Animals were selected based on a history of successful vaginal delivery and previous infant rearing experience, with estimated delivery dates separated by approximately 1 week per animal to facilitate experimental procedures. In addition to the present study of indices of brain development, these animals were used for evaluations of ETS effects on perinatal lung development, immune function, airway hyperresponsiveness through autonomic regulation, endothelial markers of mitochondrial DNA damage relevant to cardiovascular disease, and other determinations involving bone marrow, kidneys, eyes, heart, aorta, gastrointestinal tract, and reproductive organs.

To deliver ETS, we used two inhalation chambers, each with an air capacity of 3.5 m^3^, with each housing two monkeys. Aged and diluted sidestream smoke was used as a surrogate for ETS. Standardized 1R4F research cigarettes were smoked simultaneously with a single puff volume of 35 mL per cigarette and a duration of 2 sec, once per minute. Sidestream smoke from the smoldering end of each cigarette was collected and aged, and then diluted with filtered air to achieve a final particulate concentration of 1 mg/m^3^. Airflow through the system was set for 30 changes per hour, and samples were collected daily to determine the concentrations of total suspended particulates, nicotine (average, 162 μg/m^3^), and carbon monoxide (average, 4.3 ppm). These concentrations represent the high end of field measurements reported for household ETS but are within the range of what a child would experience if the caretaker is a smoker; the cloud of ETS generated around a smoker contains particulates up to 2 mg/m^3^, twice the exposure used here (Jenkins et al. 2000; U.S. Environmental Protection Agency 1992). Exposure to ETS occurred for 6 hr/day, 5 days/week, beginning at about GD50; pregnant animals in the control and postnatal exposure groups received filtered air in the same apparatus on the same schedule. All dams were allowed to give birth spontaneously, and then ETS or filtered air exposures were continued through 13 months postnatally, with the chamber containing both the mother and infant until removal of the mother at weaning (5 months of age). The group with ETS exposure limited to the postnatal period was switched from filtered air to ETS at 6 months of age and continued through 13 months. At 13 months, the offspring were anesthetized with ketamine (10 mg/kg intramuscular) and euthanized with pentobarbital (80 mg/kg intravenous). The heart was dissected and brain samples were taken from the three regions of the cerebral cortex (frontal, temporal, and occipital cortex) as well as the midbrain, using anatomical landmarks to ensure sampling of the same area from each monkey. Tissues were flash-frozen and stored at –80°C until assayed.

Each group contained both male and female offspring: four males and one female in the control group, three males and two females in the group receiving continuous ETS exposure, and three males and two females in the group receiving only postnatal ETS exposure.

In an additional set of monkeys (five males and three females in the controls, three males and five females in the ETS group), we evaluated cardiac effects elicited by continuous perinatal ETS exposure at an earlier time point (postnatal days 70–80).

### Biomarkers of neural cell development.

Tissues were thawed in 19 volumes of ice-cold 10 mM sodium–potassium phosphate buffer (pH 7.4) and homogenized with a Polytron (Brinkmann Instruments, Westbury, NY). DNA was assessed with a modified (Trauth et al. 2000) fluorescent dye-binding method (Labarca and Piagen 1980). Aliquots were diluted in 50 mM sodium phosphate, 2 M NaCl, 2 mM EDTA (pH 7.4), and sonicated briefly (Virsonic Cell Disrupter, Virtis, Gardiner, NY). Hoechst 33258 was added to a final concentration of 1 μg/mL. Samples were then read in a spectrofluorometer using an excitation wavelength of 356 nm and an emission wavelength of 458 nm and were quantitated using standards of purified DNA. The total concentration of tissue proteins was assayed from the original homogenate spectro-photometrically with bicinchoninic acid (Smith et al. 1985); in addition, we assessed the concentration of membrane proteins from the membrane preparations used for radio-ligand binding. For calculation of the ratio of membrane/total protein, the membrane protein value was averaged across the different membrane preparations.

### Thiobarbituric acid reactive species.

Lipid peroxidation was evaluated by assessment of TBARS using established techniques (Ohkawa et al. 1979). Triplicate aliquots of the same homogenate used for determination of DNA and proteins were added to an equal volume of 10% trichloroacetic acid, followed by addition of 1 volume of thiobarbituric acid reagent: 0.75% 2-thiobarbituric acid dissolved in 1 M NaOH, followed by addition of acetic acid to a final concentration of 20%. Samples were incubated for 1 hr at 95–100°C, cooled to ambient temperature, and sedimented at 3,500 × *g* for 10 min. The pellet was discarded, the supernatant solution was resedimented, and the absorbance of the final supernatant solution was determined at 532 nm. Standard curves were constructed with known concentrations of malondialdehyde that had been run through the same reaction. Values were determined relative to total protein.

### Receptor binding assays.

Cardiac receptor binding capabilities were determined by methods described previously (McMillian et al. 1983; Slotkin et al. 1987a; Song et al. 1997; Zahalka et al. 1993). Aliquots of the original tissue homogenate were sedimented at 40,000 × *g* for 15 min and were then prepared in two different ways, one for β-adrenergic receptor (βAR) binding and the other for m_2_-acetylcholine receptor (m_2_AChR) binding. For βARs, the membrane pellets were resuspended and resedimented in a buffer consisting of 125 mM sucrose, 6 mM MgCl_2_, 50 mM Tris-HCl (pH 7.5), whereas for m_2_AChR binding, we maintained the same sodium-phosphate buffer used for the original homogenization. To evaluate βAR binding, aliquots of membrane preparation were incubated with [^125^I]iodopindolol (final concentration, 67 pM), in 145 mM NaCl, 2 mM MgCl_2_, 1 mM Na ascorbate, 20 mM Tris (pH 7.5), for 20 min at room temperature in a total volume of 250 μL. Displacement of nonspecific binding was evaluated with 100 μM *d,l*-isoproterenol. Binding to m_2_AChRs was evaluated with 1 nM [^3^H]AFDX384 incubated for 60 min at room temperature in 10 mM sodium phosphate (pH 7.4), and nonspecific binding was evaluated with 1 μM atropine.

### Data analysis.

Data are presented as means and SEs. The effects of ETS exposure were first evaluated by global analysis of variance (ANOVA; data log-transformed because of heterogeneous variance) incorporating the three different treatments (control, continuous perinatal ETS, postnatal ETS 6–13 months), the various regions, and the repeated measures representing the biomarkers of neural cell development: DNA concentration, total protein/DNA ratio, and membrane/total protein ratio. Because this initial test indicated a significant difference of treatment effects according to the type of measurement, we used lower order ANOVAs (treatment, region) to assess the effects separately for each measure. Finally, where the lower order test indicated an interaction of treatment with region, separate post hoc analyses (Fisher’s protected least significant difference) were undertaken to determine the effects of ETS exposure on each individual region; in the absence of an interaction, only the main effect of ETS was reported. Similarly, TBARS were assessed initially with a two-factor ANOVA (treatment, region), and cardiac receptor binding studies were first evaluated by ANOVA incorporating treatment and receptor type (βAR, m_2_AChR). Significance for all tests was assumed at *p* < 0.05.

## Results

Prepartum ultrasonography performed at GD40, GD90, GD120, and GD150 revealed no significant differences in fetal growth between those exposed to ETS or those exposed to filtered air. Similarly, the ETS group showed normal weights and other somatic indices of gestational age at birth, and there were no effects on growth through 13 months postnatal age (not shown).

**Figure 1:**
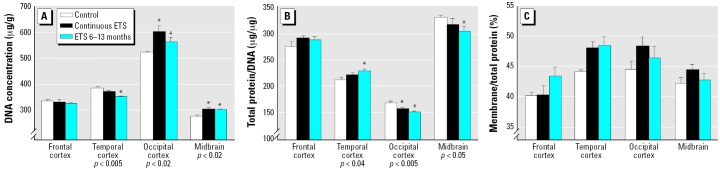
Effects of ETS exposure on biomarkers of neural cell development: (*A*) DNA concentration (ANOVA: treatment, *p* < 0.01; treatment × region, *p* < 0.0001). (*B*) Total protein/DNA ratio (ANOVA: treatment × region, *p* < 0.004). (*C*) Membrane/total protein ratio (ANOVA: treatment, *p* < 0.008; there was no treatment × region interaction); the main effect of each ETS treatment in (*C*) is as follows: continuous ETS, *p* < 0.005; ETS 6–13 months, *p* < 0.008. *Individual values for which the ETS groups differ from the corresponding control. These were not evaluated in (*C*) because of the absense of a treatment × region interaction.

At 13 months of age, there were no differences in body weights among the three groups (control, 2.3 ± 0.1 kg; continuous ETS, 2.2 ± 0.2 kg; ETS 6–13 months, 2.3 ± 0.2 kg), nor were there differences in general health or activity. Nevertheless, ANOVA across the three biomarkers of neural cell development indicated highly significant differences among the three groups (*p* < 0.0008) that depended both upon the specific measure and brain region (treatment × measure × region, *p* < 0.0002). Accordingly, we subdivided the assessments into the three different developmental indices and reevaluated the main treatment effects and regional specificity. The DNA concentration, an index of cell packing density, showed regionally selective changes elicited by ETS exposure (Figure 1A). Although values were unaffected in the frontal cortex, both the occipital cortex and midbrain displayed significant increases after either continuous ETS exposure or ETS exposure restricted to the postnatal 6–13 month period. In contrast, values tended to be reduced in the temporal cortex, achieving statistical significance with the postnatal exposure group.

Both indices of cell size also displayed ETS-induced differences. For the total protein/DNA ratio, the values were reciprocally related to the change in DNA concentration. Accordingly, reductions were seen in the occipital cortex and midbrain, whereas an increase was obtained in the temporal cortex (Figure 1B). The membrane/total protein ratio showed overall increases that were not regionally selective but that were statistically significant both for continuous ETS exposure and for the group receiving only postnatal exposure (Figure 1C).

**Figure 2:**
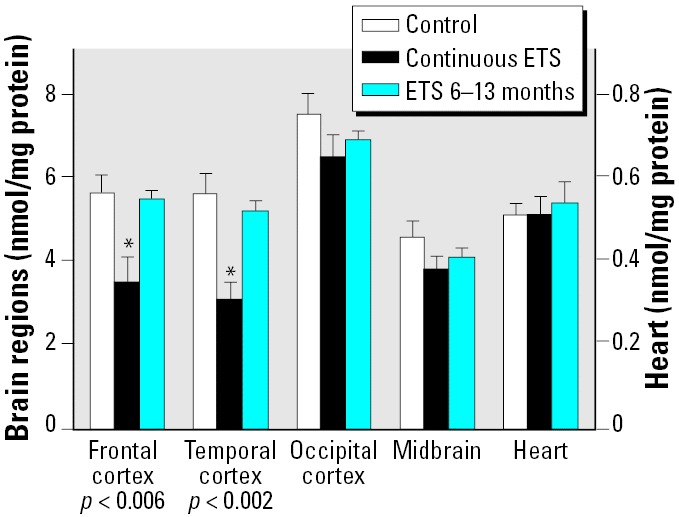
Effects of ETS exposure on TBARS in brain regions and heart (note different scales). ANOVA across all treatments and tissues: treatment, *p* < 0.0001; treatment × tissue, *p* < 0.004. Lower order ANOVAs for each tissue are shown within the figure. *Individual values for which the ETS groups differ from the corresponding control.

In contrast to the similarity of effects of continuous perinatal ETS exposure and postnatal exposure on neural cell development bio-markers, there were radically different effects on TBARS (Figure 2). The continuous ETS group showed marked reductions in TBARS in the frontal cortex and temporal cortex, without significant effects in the other regions or in the heart. In contrast, when ETS exposure occurred postnatally from 6–13 months, there were no significant differences in TBARS. The absence of effects in the heart, a tissue that, like the brain, has high oxygen demand, could imply that only the brain is targeted by ETS exposure, or alternatively that the heart may show similar effects but with a different temporal relationship. To distinguish these two possibilities, we performed an additional study with continuous ETS exposure, but conducting the evaluations earlier, on postnatal days 70–80. Under these circumstances, we obtained the same robust decrease in TBARS in the heart that we observed later in the brain (control, 1.71 ± 0.4 nmol/mg protein; ETS, 0.88 ± 0.11 nmol/mg protein; *p* < 0.008).

**Figure 3:**
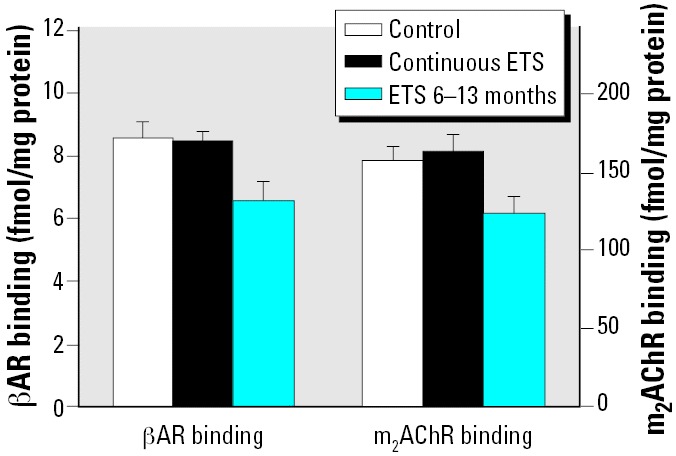
Effects of ETS exposure on cardiac βAR and m_2_AChR binding (note different scales); ANOVA: treatment, *p* < 0.004. There was no interaction of treatment × receptor type; the only main treatment effect is ETS 6–13 months (*p* < 0.003). There were no significant differences in the concentration of membrane proteins.

Earlier studies in rodents, using either nicotine or ETS exposure, indicated down-regulation and/or desensitization of cardiac autonomic receptors whose activity influences oxidative demand (Joseph et al. 2002; Navarro et al. 1990; Remondino et al. 2003; Slotkin et al. 1999, 2001). Accordingly, we assessed effects on both cardiac βAR and m_2_AChR binding with both the continuous perinatal exposure and postnatal exposure models (Figure 3). Although continuous exposure had no significant effect, both receptor types were down-regulated in the group where ETS exposure was restricted to the postnatal period of 6–13 months.

## Discussion

Perinatal or postnatal ETS exposure elicited two characteristic patterns of neural cellular effects, both of which resemble earlier findings for effects of prenatal nicotine exposure in rodents (Levin and Slotkin 1998; Roy et al. 1998, 2002; Roy and Sabherwal 1994, 1998; Slotkin 1998, 2004; Slotkin et al. 1987b). In the occipital cortex and midbrain, there were smaller cells (reduced total protein/DNA ratio) and a corresponding increase in cell packing density (DNA concentration), features that are likely to reflect neuronal damage and “reactive gliosis,” that is, replacement with smaller, glial cells (O’Callaghan 1988, 1993; Roy et al. 1998, 2002; Roy and Sabherwal 1994, 1998). In contrast, in the temporal cortex, we found a reduction in the total number of cells (reduced DNA) with hypertrophy of the remaining cells (increased total protein/DNA ratio), changes indicative of cell loss with perikaryal swelling (Roy et al. 2005). Superimposed on these two patterns, we also found an overall increase in the membrane/total protein ratio, which is compatible either with smaller cells (higher surface-to-volume ratio) or with increased neuritic sprouting. Given the disparate underlying regional patterns for the other two markers, the first explanation is likely to be true for the occipital cortex and midbrain, whereas the latter is more probable for the temporal cortex: reactive sprouting is typical after damage to developing nerve terminals or projections (Kostrzewa and Jacobowitz 1974) and, again, has been found for the effects of prenatal nicotine exposure in rodents (Navarro et al. 1988). These neurochemical inferences point to the need for detailed, quantitative morphologic investigations of ETS effects on primate development paralleling those done for nicotine in rodent models (Roy et al. 1998, 2002; Roy and Sabherwal 1994, 1998), and the present results provide the necessary guidance as to which regions should be evaluated and what types of changes are likely to be found.

In addition to regional selectivity, there were two other notable features of ETS-induced alterations in neurochemistry. First, although the changes were statistically significant, not surprisingly, the effects were smaller in magnitude than those associated with direct nicotine administration (Levin and Slotkin 1998; Slotkin 1998, 2004). Given that ETS delivers higher levels of oxidative free radicals than does just the administration of nicotine (Huang et al. 2005), our results imply that the role of nicotine in adverse neurobehavioral outcomes is primary; indeed, as discussed below, nicotine-induced damage may actually limit the contributions of oxidative injury. Nevertheless, as seen here for different cortical regions, heterogeneity of the effects is likely to reduce measured differences by diluting highly affected nuclei or neuron types with larger amounts of unaffected subregions. Consequently, biochemical examinations of even broader regional groupings may give false negative results because of opposing changes in different subregions (Gospe et al. 1996). Even here, with subregional dissection into the frontal, temporal, and occipital cortex, we are still incorporating heterogeneous layers and nuclei, which means that significant, small changes imply much larger focal effects that are likely to be identified by quantitative morphology. Indeed, with prenatal nicotine exposure in rats, we have already shown distinct targeting of different types of neurons even within a single layer of the somatosensory cortex or in specific zones of the hippocampus (Roy and Sabherwal 1994, 1998; Roy et al. 2002).

The second unexpected feature of the effects of ETS was that both continuous and postnatal exposure produced neurochemical changes that were similar in regional selectivity and magnitude, despite the obvious, major differences in exposure period and duration. Translated to human ETS exposure, this finding points out the importance of reducing the exposure of young children to tobacco smoke in the home or in child care settings. However, our results also pose a conundrum: how can continuous perinatal exposure give the same net effect as exposure restricted to the postnatal period of 6–13 months of age? It is highly unlikely that damage to the developing brain occurs only with postnatal exposure, given the known effects of prenatal nicotine on brain development (Levin and Slotkin 1998; Slotkin 1998, 2004; Slotkin et al. 2005). Alternatively, the effects of continuous perinatal exposure may be greater than those of postnatal ETS, with the differences masked by the limits of resolution imposed by regional heterogeneity; in that case, detailed morphologic studies will again reveal the disparities between the two exposure paradigms. However, our results for lipid peroxidation also point to the possibility that some factors operate to constrain the degree of these specific types of cellular damage. Surprisingly, perinatal ETS exposure reduced TBARS in cortical subregions, rather than evoking the expected increase, thus suggesting an enhancement of antioxidant defenses in the exposed offspring. This result is in keeping with a recent study of human maternal and cord blood, which similarly found an increase in antioxidant molecules with active smoking during pregnancy and smaller changes in the same direction with ETS exposure (Fayol et al. 2005). Here, we found evidence that prenatal ETS exposure programs antioxidant responses that limit the additional effects of postnatal ETS: TBARS were reduced with the perinatal exposure model but not with postnatal exposure, despite the fact that both groups received equivalent ETS for the 7 months preceding the tissue sampling at 13 months of age. It is likely that programming of defense mechanisms is still going on in the neonatal period, albeit at a much lower level than with prenatal exposure, because we did not find an elevation in TBARS in the postnatal ETS group. In contrast, nicotine administered by itself to older animals produces an increase in TBARS in a variety of brain regions, even at nicotine doses simulating ETS exposure (Qiao et al. 2005), whereas much higher doses in the fetus do not (Slotkin et al. 2005). Although we did not evaluate which specific mechanisms contribute to the net reductions in TBARS, it is important to note that some of the factors may actually not be beneficial. During development, a mild degree of oxidative stress is required for the appropriate timing of neuronal cell differentiation (Katoh et al. 1997), so oxidative stress from ETS exposure and the adaptive changes in defense mechanisms are both likely to preempt this natural signal. Furthermore, a number of the known, neurotoxic effects of nicotine on brain development are themselves liable to reduce oxidative damage. Nicotine actually protects developing neurons from the effects of other oxidative molecules (Guan et al. 2003; Qiao et al. 2005). In addition, the developmental neurotoxicity of nicotine produces changes that promote resistance to oxidative stress, including marked reductions in synaptic development and activity, and the replacement of damaged neurons with glia, cells that possess major antioxidant pathways (Levin and Slotkin 1998; Roy et al. 2002; Slotkin 1998, 2004; Slotkin et al. 2005; Tanaka et al. 1999). Specifically, prenatal nicotine exposure grossly reduces neonatal activity of nerve pathways using catecholamine neuro-transmitters (Levin and Slotkin 1998; Slotkin 1998, 2004), which are strongly oxidative (Olanow and Arendash 1994). Accordingly, the primary neurotoxic effects of nicotine may limit the apparent contribution of oxidative damage to the net neurobehavioral effects of ETS, so looking at lipid peroxidation alone may be misleading without considering the whole picture.

Unlike the effects of ETS on TBARS in the brain, we did not find significant reductions in the heart after perinatal ETS exposure, nor did postnatal ETS produce an effect. These results indicate either that the heart displays a different critical period for the programming of antioxidant defenses, or alternatively that the timetable for appearance and disappearance of the effect might be different. In fact, when we examined lipid peroxidation at an earlier time point 2–3 months after birth, we were able to demonstrate a significant reduction in cardiac TBARS in the perinatal exposure group, implying that the effects were present but disappeared by the later sampling at 13 months of age. Similarly, then, brain regions that did not display a significant decrease at 13 months may not in fact be spared from the effects but may simply show a more rapid return to normal oxidative status. The temporal dichotomy is a reflection of the fact that TBARS measurements take a momentary “snapshot” of lipid peroxidation rather than representing long-term damage, whereas neural cell biomarkers provide a much longer integrative time frame.

The results in the heart also provide confirmation that the protection from oxidative stress comprises alterations that actually reflect functional loss, evidenced by the reductions in βARs and m_2_AChRs. Cardiac βAR overstimulation evokes oxidative stress, leading to myocyte apoptosis (Remondino et al. 2003), whereas βARs protect neurons (Sarker et al. 2000) and show no down-regulation by developmental ETS exposure (Slotkin et al. 2005). In turn, cardiac m_2_AChRs may be reduced as a compensation to maintain the balance of autonomic input or, alternatively, may be specifically down-regulated because of their similar involvement in oxidative stress (Joseph et al. 2002). Indeed, in rats with ETS exposure, the degree of cardiac m_2_AChR down-regulation exceeds that of βARs (Slotkin et al. 2001). Again, there may be a specific role for nicotine in these potentially maladaptive responses: by itself, prenatal nicotine exposure leads to decrements in cardiac βAR function (Navarro et al. 1990).

In summary, our findings show that perinatal or postnatal ETS exposure in primates elicits changes in brain cell development akin to those found for either prenatal nicotine exposure or perinatal ETS exposure in rodents (Gospe et al. 1996; Levin and Slotkin 1998; Navarro et al. 1988; Slotkin 1998, 2004) as well as for prenatal nicotine in monkeys (Slotkin et al. 2005). This reinforces a mechanistic connection between nicotine as a specific contributor to the adverse neurobehavioral effects of developmental ETS exposure and supports the use of nicotine metabolite measurements in fetuses and children as an appropriate predictor of outcome (Eliopoulos et al. 1996; Fried et al. 1995; Jauniaux et al. 1999; Kohler et al. 1999; Ostrea et al. 1994). Equally significant, we found that postnatal ETS produces effects very similar to those achieved with continuous prenatal and postnatal exposure, buttressing the importance of restricting or eliminating exposure in young children. Finally, although ETS exposure also elicits signs of chronic oxidative stress, demonstration of a specific role of this mechanism in brain damage remains elusive, confounded by adaptive mechanisms and perhaps most of all by the underlying damage caused by nicotine. Indeed, for prenatal exposure, attempts to offset oxidative damage by dietary supplementation with antioxidants may actually worsen nicotine-related neurodevelopmental damage by secondary pharmacokinetic effects that increase nicotine concentrations in the fetal compartment (Slotkin et al. 2005), indicating the danger of focusing on oxidative damage as a primary mechanism rather than on the net neurotoxic outcome of all ETS components.

## References

[b1-ehp0114-000034] Bell JM, Whitmore WL, Queen KL, Orband-Miller L, Slotkin TA (1987). Biochemical determinants of growth sparing during neonatal nutritional deprivation or enhancement: ornithine decarboxylase, polyamines, and macromolecules in brain regions and heart. Pediatr Res.

[b2-ehp0114-000034] Bhagwat SV, Vijayasarathy C, Raza H, Mullick J, Avadhani NG (1998). Preferential effects of nicotine and 4-(*N*-methyl-*N*-nitrosamino)-1-(3-pyridyl)-1-butanone on mitochondrial glutathione *S*-transferase A4-4 induction and increased oxidative stress in the rat brain. Biochem Pharmacol.

[b3-ehp0114-000034] Dunn A Zeise L 1997. Health Effects of Exposure to Environmental Tobacco Smoke. Sacramento, CA:California Environmental Protection Agency. 10.1136/tc.6.4.346 PMC1759599 9583639

[b4-ehp0114-000034] Eliopoulos C, Klein J, Chitayat D, Greenwald M, Koren G (1996). Nicotine and cotinine in maternal and neonatal hair as markers of gestational smoking. Clin Invest Med.

[b5-ehp0114-000034] Eskenazi B, Trupin LS (1995). Passive and active maternal smoking during pregnancy, as measured by serum cotinine, and postnatal smoke exposure. 2. effect on neurodevelopment at age 5 years. Am J Epidemiol.

[b6-ehp0114-000034] Fayol L, Gulian JM, Dalmasso C, Calaf R, Simeoni U, Millet V (2005). Antioxidant status of neonates exposed in utero to tobacco smoke. Biol Neonate.

[b7-ehp0114-000034] Fried PA, O’Connell CM, Watkinson B (1992). 60- and 72-month follow-up of children prenatally exposed to marijuana, cigarettes, and alcohol: cognitive and language assessment. J Dev Behav Pediatr.

[b8-ehp0114-000034] Fried PA, Perkins SL, Watkinson B, McCartney JS (1995). Association between creatinine-adjusted and unadjusted urine cotinine values in children and the mother’s report of exposure to environmental tobacco smoke. Clin Biochem.

[b9-ehp0114-000034] Fried PA, Watkinson B, Gray R (1998). Differential effects on cognitive functioning in 9- to 12-year olds prenatally exposed to cigarettes and marihuana. Neurotoxicol Teratol.

[b10-ehp0114-000034] Fried PA, Watkinson B, Gray R (2003). Differential effects on cognitive functioning in 13- to 16-year-olds prenatally exposed to cigarettes and marihuana. Neurotoxicol Teratol.

[b11-ehp0114-000034] Gitto E, Reiter RJ, Karbownik M, Tan DX, Gitto P, Barberi S (2002). Causes of oxidative stress in the pre- and perinatal period. Biol Neonate.

[b12-ehp0114-000034] Gospe SM, Zhou SS, Pinkerton KE (1996). Effects of environmental tobacco smoke exposure *in utero* and/or postnatally on brain development. Pediatr Res.

[b13-ehp0114-000034] Guan ZZ, Yu WF, Nordberg A (2003). Dual effects of nicotine on oxidative stress and neuroprotection in PC12 cells. Neurochem Intl.

[b14-ehp0114-000034] Gupta RC (2004). Brain regional heterogeneity and toxicological mechanisms of organophosphates and carbamates. Toxicol Mech Methods.

[b15-ehp0114-000034] Huang MF, Lin WL, Ma YC (2005). A study of reactive oxygen species in mainstream of cigarette. Indoor Air.

[b16-ehp0114-000034] Hutchison SJ, Glantz SA, Zhu BQ, Sun YP, Chou TM, Chatterjee K (1998). In-utero and neonatal exposure to secondhand smoke causes vascular dysfunction in newborn rats. J Am Coll Cardiol.

[b17-ehp0114-000034] James SJ, Slikker W, Melnyk S, New E, Pogribna M, Jernigan S (2005). Thimerosal neurotoxicity is associated with glutathione depletion: protection with glutathione precursors. Neurotoxicology.

[b18-ehp0114-000034] Jauniaux E, Gulbis B, Acharya G, Thiry P, Rodeck C (1999). Maternal tobacco exposure and cotinine levels in fetal fluids in the first half of pregnancy. Obstet Gynecol.

[b19-ehp0114-000034] Jenkins RA Guerin MR Tomkins BA 2000. The Chemistry of Environmental Tobacco Smoke: Composition and Measurement. 2nd ed. Boca Raton, FL:Lewis Publishers.

[b20-ehp0114-000034] Joseph JA, Fisher DR, Strain J (2002). Muscarinic receptor subtype determines vulnerability to oxidative stress in COS-7 cells. Free Radic Biol Med.

[b21-ehp0114-000034] Katoh S, Mitsui Y, Kitani K, Suzuki T (1997). Hyperoxia induces the differentiated neuronal phenotype of PC12 cells by producing reactive oxygen species. Biochem Biophys Res Commun.

[b22-ehp0114-000034] Kohler E, Sollich V, Schuster R, Thal W (1999). Passive smoke exposure in infants and children with respiratory tract diseases. Human Exp Toxicol.

[b23-ehp0114-000034] Kostrzewa R, Jacobowitz DM (1974). Pharmacological actions of 6-hydroxydopamine. Pharmacol Rev.

[b24-ehp0114-000034] Labarca C, Piagen K (1980). A simple, rapid, and sensitive DNA assay procedure. Anal Biochem.

[b25-ehp0114-000034] Levin ED Slotkin TA 1998. Developmental neurotoxicity of nicotine. In: Handbook of Developmental Neurotoxicology (Slikker W, Chang LW, ed). San Diego:Academic Press, 587–615.

[b26-ehp0114-000034] Makin J, Fried PA, Watkinson B (1991). A comparison of active and passive smoking during pregnancy: long-term effects. Neurotoxicol Teratol.

[b27-ehp0114-000034] McMillian MK, Schanberg SM, Kuhn CM (1983). Ontogeny of rat hepatic adrenoceptors. J Pharmacol Exp Ther.

[b28-ehp0114-000034] National Research Council 1996. Guide for the Care and Use of Laboratory Animals. Washington, DC:National Academy Press.

[b29-ehp0114-000034] Navarro HA, Mills E, Seidler FJ, Baker FE, Lappi SE, Tayyeb MI (1990). Prenatal nicotine exposure impairs β-adrenergic function: persistent chronotropic subsensitivity despite recovery from deficits in receptor binding. Brain Res Bull.

[b30-ehp0114-000034] Navarro HA, Seidler FJ, Whitmore WL, Slotkin TA (1988). Prenatal exposure to nicotine *via* maternal infusions: effects on development of catecholamine systems. J Pharmacol Exp Ther.

[b31-ehp0114-000034] Newman MB, Arendash GW, Shytle RD, Bickford PC, Tighe T, Sanberg PR (2002). Nicotine’s oxidative and antioxidant properties in CNS. Life Sciences.

[b32-ehp0114-000034] O’Callaghan JP (1988). Neurotypic and gliotypic proteins as biochemical markers of neurotoxicity. Neurotoxicol Teratol.

[b33-ehp0114-000034] O’Callaghan JP (1993). Quantitative features of reactive gliosis following toxicant-induced damage of the CNS. Ann NY Acad Sci.

[b34-ehp0114-000034] Ohkawa H, Ohishi N, Yagi K (1979). Assay for lipid peroxides in animal tissues by thiobarbituric acid reaction. Anal Biochem.

[b35-ehp0114-000034] Ohtsuka K, Suzuki T (2000). Roles of molecular chaperones in the nervous system. Brain Res Bull.

[b36-ehp0114-000034] Olanow CW, Arendash GW (1994). Metals and free radicals in neurodegeneration. Curr Opin Neurol.

[b37-ehp0114-000034] Ostrea EM, Knapp DK, Romero A, Montes M, Ostrea AR (1994). Meconium analysis to assess fetal exposure to nicotine by active and passive maternal smoking. J Pediatr.

[b38-ehp0114-000034] Qiao D, Seidler FJ, Abreu-Villaça Y, Tate CA, Cousins MM, Slotkin TA (2004). Chlorpyrifos exposure during neurulation: cholinergic synaptic dysfunction and cellular alterations in brain regions at adolescence and adulthood. Dev Brain Res.

[b39-ehp0114-000034] Qiao D, Seidler FJ, Slotkin TA (2005). Oxidative mechanisms contributing to the developmental neurotoxicity of nicotine and chlorpyrifos. Toxicol Appl Pharmacol.

[b40-ehp0114-000034] Qiao D, Seidler FJ, Tate CA, Cousins MM, Slotkin TA (2003). Fetal chlorpyrifos exposure: adverse effects on brain cell development and cholinergic biomarkers emerge postnatally and continue into adolescence and adulthood. Environ Health Perspect.

[b41-ehp0114-000034] Remondino A, Kwon SH, Communal C, Pimentel DR, Sawyer DB, Singh K (2003). β-Adrenergic receptor-stimulated apoptosis in cardiac myocytes is mediated by reactive oxygen species/c-Jun NH2-terminal kinase-dependent activation of the mitochondrial pathway. Circ Res.

[b42-ehp0114-000034] Rodier PM (1988). Structural-functional relationships in experimentally induced brain damage. Prog Brain Res.

[b43-ehp0114-000034] Roy TS, Andrews JE, Seidler FJ, Slotkin TA (1998). Nicotine evokes cell death in embryonic rat brain during neurulation. J Pharmacol Exp Ther.

[b44-ehp0114-000034] Roy TS, Sabherwal U (1994). Effects of prenatal nicotine exposure on the morphogenesis of somatosensory cortex. Neurotoxicol Teratol.

[b45-ehp0114-000034] Roy TS, Sabherwal U (1998). Effects of gestational nicotine exposure on hippocampal morphology. Neurotoxicol Teratol.

[b46-ehp0114-000034] Roy TS, Seidler FJ, Slotkin TA (2002). Prenatal nicotine exposure evokes alterations of cell structure in hippocampus and somatosensory cortex. J Pharmacol Exp Ther.

[b47-ehp0114-000034] Roy TS, Sharma V, Seidler FJ, Slotkin TA (2005). Quantitative morphological assessment reveals neuronal and glial deficits in hippocampus after a brief subtoxic exposure to chlorpyrifos in neonatal rats. Dev Brain Res.

[b48-ehp0114-000034] Sarker KP, Uchimura T, Nakajima T, Sorimachi M, Kitajima I, Maruyama I (2000). Epinephrine prevents nitric oxide/per-oxynitrite induced apoptosis of neuronal cells through β-adrenergic receptor activation. Neurosci Res Commun.

[b49-ehp0114-000034] Slotkin TA (1998). Fetal nicotine or cocaine exposure: which one is worse?. J Pharmacol Exp Ther.

[b50-ehp0114-000034] Slotkin TA (2004). Cholinergic systems in brain development and disruption by neurotoxicants: nicotine, environmental tobacco smoke, organophosphates. Toxicol Appl Pharmacol.

[b51-ehp0114-000034] Slotkin TA, Epps TA, Stenger ML, Sawyer KJ, Seidler FJ (1999). Cholinergic receptors in heart and brainstem of rats exposed to nicotine during development: implications for hypoxia tolerance and perinatal mortality. Dev Brain Res.

[b52-ehp0114-000034] Slotkin TA, Orband-Miller L, Queen KL (1987a). Development of [^3^H]nicotine binding sites in brain regions of rats exposed to nicotine prenatally *via* maternal injections or infusions. J Pharmacol Exp Ther.

[b53-ehp0114-000034] Slotkin TA, Orband-Miller L, Queen KL, Whitmore WL, Seidler FJ (1987b). Effects of prenatal nicotine exposure on biochemical development of rat brain regions: maternal drug infusions *via* osmotic minipumps. J Pharmacol Exp Ther.

[b54-ehp0114-000034] Slotkin TA, Persons D, Slepetis RJ, Taylor D, Bartolome J (1984). Control of nucleic acid and protein synthesis in developing brain, kidney, and heart of the neonatal rat: effects of α-difluoromethylornithine, a specific, irreversible inhibitor of ornithine decarboxylase. Teratology.

[b55-ehp0114-000034] Slotkin TA, Pinkerton KE, Auman JT, Qiao D, Seidler FJ (2002). Perinatal exposure to environmental tobacco smoke upregulates nicotinic cholinergic receptors in monkey brain. Dev Brain Res.

[b56-ehp0114-000034] Slotkin TA, Pinkerton KE, Garofolo MC, Auman JT, McCook EC, Seidler FJ (2001). Perinatal exposure to environmental tobacco smoke induces adenylyl cyclase and alters receptor-mediated signaling in brain and heart of neonatal rats. Brain Res.

[b57-ehp0114-000034] Slotkin TA, Pinkerton KE, Seidler FJ (2000). Perinatal exposure to environmental tobacco smoke alters cell signaling in a primate model: autonomic receptors and the control of adenylyl cyclase activity in heart and lung. Dev Brain Res.

[b58-ehp0114-000034] Slotkin TA, Seidler FJ, Qiao D, Aldridge JE, Tate CA, Cousins MM (2005). Effects of prenatal nicotine exposure on primate brain development and attempted amelioration with supplemental choline or vitamin C: neurotransmitter receptors, cell signaling and cell development biomarkers in fetal brain regions of rhesus monkeys. Neuropsychopharmacology.

[b59-ehp0114-000034] Smith PK, Krohn RI, Hermanson GT, Mallia AK, Gartner FH, Provenzano MD (1985). Measurement of protein using bicinchoninic acid. Anal Biochem.

[b60-ehp0114-000034] Song X, Seidler FJ, Saleh JL, Zhang J, Padilla S, Slotkin TA (1997). Cellular mechanisms for developmental toxicity of chlorpyrifos: targeting the adenylyl cyclase signaling cascade. Toxicol Appl Pharmacol.

[b61-ehp0114-000034] Tanaka J, Toku K, Zhang B, Isihara K, Sakanaka M, Maeda N (1999). Astrocytes prevent neuronal death induced by reactive oxygen and nitrogen species. Glia.

[b62-ehp0114-000034] Trauth JA, Seidler FJ, Slotkin TA (2000). An animal model of adolescent nicotine exposure: effects on gene expression and macromolecular constituents in rat brain regions. Brain Res.

[b63-ehp0114-000034] U.S. Environmental Protection Agency 1992. Respiratory Health Effects of Passive Smoking: Lung Cancer and Other Disorders. Washington, DC:Office of Research and Development, U.S. Environmental Protection Agency.

[b64-ehp0114-000034] Wakschlag LS, Pickett KE, Cook E, Benowitz NL, Leventhal BL (2002). Maternal smoking during pregnancy and severe antisocial behavior in offspring: a review. Am J Public Health.

[b65-ehp0114-000034] Walker A, Rosenberg M, Balaban-Gil K (1999). Neuro-developmental and neurobehavioral sequelae of selected substances of abuse and psychiatric medications in utero. Child Adolesc Psychiat Clin North Am.

[b66-ehp0114-000034] Weitzman M, Byrd RS, Aligne CA, Moss M (2002). The effects of tobacco exposure on children’s behavioral and cognitive functioning: implications for clinical and public health policy and future research. Neurotoxicol Teratol.

[b67-ehp0114-000034] Winick M, Noble A (1965). Quantitative changes in DNA, RNA and protein during prenatal and postnatal growth in the rat. Dev Biol.

[b68-ehp0114-000034] Witschi H, Joad JP, Pinkerton KE (1997). The toxicology of environmental tobacco smoke. Annu Rev Pharmacol Toxicol.

[b69-ehp0114-000034] Yildiz D, Liu YS, Ercal N, Armstrong DW (1999). Comparison of pure nicotine- and smokeless tobacco extract-induced toxicities and oxidative stress. Arch Environ Contam Toxicol.

[b70-ehp0114-000034] Zahalka EA, Seidler FJ, Yanai J, Slotkin TA (1993). Fetal nicotine exposure alters ontogeny of M_1_-receptors and their link to G-proteins. Neurotoxicol Teratol.

